# Comparison of empathy profiles of medical students at the start and in the advanced clinical phase of their training

**DOI:** 10.3205/zma001662

**Published:** 2024-02-15

**Authors:** Susanne Schrötter, Peter Kropp, Britta Müller

**Affiliations:** 1 Universitätsmedizin Rostock, Institut für Medizinische Psychologie und Medizinische Soziologie, Rostock, Germany

**Keywords:** empathy, emotion regulation, medical studies, patient satisfaction, burnout

## Abstract

**Background:**

The National Competence Based Catalogue of Learning Objectives for Undergraduate Medical Education (NKLM) cites empathy as a basic competence for medical doctors. Based on a multidimensional concept of clinical empathy, empathy profiles of medical students at the start of their training and in the 9^th^ semester were identified and compared in order to draw conclusions for the conception of effective course offers.

**Method:**

Using the Saarbrücker Personality Questionnaire on Empathy (SPF-IRI), self-rated empathy was recorded in a cross-sectional study of medical students (1^st^ semester: N=192/9^th^ semester: N=221). Two Stage Clustering was performed for data analysis.

**Result:**

Three empathy profiles which could be meaningfully delineated by content were identified: 1. reflected, functional empathy, 2. unreflected, burdensome empathy and 3. distancing and avoidance. Students in the 9^th^ semester mostly tended toward unreflected, burdensome empathy. Only one-third appeared capable of feeling empathy with patients while at the same time adequately regulating their own emotions and thus protecting themselves from emotional overload.

**Conclusion:**

An adequately reflected and functional empathy among medical students can neither be assumed at the start of their training, nor do existing course offers appear to provide sufficient training for this. Empathy should thus be implemented as a competence which needs to be promoted over the entire course of study. Emotion regulation plays a key role.

## Zusammenfassung

**Hintergrund:**

Der Nationale Kompetenzbasierte Lernzielkatalog Medizin (NKLM) formuliert Empathie als grundlegende ärztliche Kompetenz. Basierend auf einem multidimensionalen Konzept ärztlicher Empathie wurden Empathie-Profile Medizinstudierender zu Studienbeginn und im 9. Semester identifiziert und verglichen, um Schlussfolgerungen für die Konzeption effektiver Lehrangebote generieren zu können.

**Methode:**

In einer Querschnittstudie wurde mittels Saarbrücker Persönlichkeitsfragebogen zu Empathie (SPF-IRI) bei Medizinstudierenden (1. Semester: N=192/9. Semester: N=221) die selbstbeurteilte Empathie erhoben. Für die Datenanalyse wurde ein Two Stage Clustering durchgeführt.

**Ergebnis:**

Drei inhaltlich sinnvoll abgrenzbare Empathie-Profile wurden identifiziert: 1. reflektierte, funktionale Empathie, 2. unreflektierte, belastende Empathie und 3. Distanzierung und Vermeidung. Studierende im 9. Semester zeigten überwiegend die Neigung zu unreflektierter, belastender Empathie. Nur ein Drittel schien fähig zu sein, sich empathisch auf Patient*innen einzulassen und dabei gleichzeitig die eigenen Emotionen angemessen regulieren und sich so vor emotionaler Überlastung schützen zu können.

**Schlussfolgerung:**

Eine ausreichend reflektierte und funktionale Empathie kann bei Medizinstudierenden weder zu Studienbeginn vorausgesetzt werden, noch scheinen bestehende Lehrangebote diese hinreichend auszubilden. Empathie sollte daher als zu stärkende Kompetenz im gesamten Studienverlauf implementiert werden. Dabei spielt die Emotionsregulation eine Schlüsselrolle.

## 1. Introduction

### 1.1. Background

The National Competence Based Catalogue of Learning Objectives for Undergraduate Medical Education (NKLM) cites empathy as a basic superordinate competence for doctors, which should be continuously promoted over the entire course of medical training [https://nklm.de/zend/menu]. Its high relevance for the quality of medical care and patient satisfaction has been empirically proven [1], [2], [3], [4], [5]. The observed improvement in therapy outcomes is attributed primarily to the creation of confidence between doctor and patient [6], [7], [8], [9]. A connection between empathy and job satisfaction [10], [11], resilience [12], burnout prophylaxis and well-being [11], [13], [14], [15], [16] among doctors has also been demonstrated in numerous studies. Moreover, empathy is also considered beneficial with respect to the capacity for teamwork, leadership competence and constructive conflict behaviour [17], [18], [19] – desirable competencies for cooperation in the complex interprofessional teams customarily found in the clinical setting. 

At the same time, the doctor-in-training is expected to show professional distance [https://nklm.de/zend/menu] and is warned about sympathy, since that impairs clinical neutrality, the objectivity of diagnostics and treatment, and personal resilience [20]. A connection between empathy and burnout due to high emotional demands of the profession has also been the subject of empirical studies, both for doctors [12], [21], [22], [23] and medical students [24], [25], [26], [27]. It is assumed that an unreflected flow of empathic emotions coupled with the potentially high frequency of emotional situations in everyday routine increases the experience of stress. Moreover, there is a clear tendency in research literature to attribute more professionality to technically-experienced, rational and emotionally-distanced doctors than to sympathetic doctors [3], [6], [28], [29], [30], [31], [32], [33]. This results in a field of tension which is reflected in the discussion about the definition of clinical empathy. 

### 1.2. Clinical empathy 

A mostly cognitive understanding of doctors’ empathy is widespread in research on medical education [34]. It assumes an objective, rational and intellectual process of understanding the experiences, feelings and viewpoints of patients, which is frequently called perspective taking [35], coupled with the competence to communicate this understanding in a supportive manner [5], [32], [36], [37]. On the other hand, emotional involvement of the doctor, as an affective component of empathy, is taken to be detrimental to good clinical practice and is rejected [3], [20], [31], [38]. 

Contrary to this, our study is a multidimensional approach which views empathy as a complex construct combining affective and cognitive components with the capability of being able to regulate empathically elicited emotions and thus influence the empathic behaviour of the moment [39], [40]. It offers the possibility of viewing the various components of empathy separately and thus reflect on effective and satisfactory behaviour for a doctor which includes both distance from and empathy for patients – and yet protects against emotional overload. Gleichgerrcht and Decety differentiate four interacting dimensions:

1. empathic arousal (sharing the affective state of another),
2. empathic understanding (formation of an explicit mental representation of the emotional state of another person),
3. empathic concern (feeling other-oriented emotions for someone in need as motivation to maintain the interaction) and
4. emotion regulation (the control of one’s own emotions and motivation) [13].

Affective and cognitive dimensions here are thus closely connected to one another and exert reciprocal influence [39], [40], [41], [42]. It is assumed that cognitive understanding is always coupled with emotional resonance – albeit to various extents [10]. Whether the doctor’s sharing or reflecting the emotions leads to other-focussed empathic concern or to self-focussed emotional distress and overburdening (personal distress) [35], [43], is determined by self-other-differentiation [44]. According to numerous studies, the capability of differentiating between one’s own and others’ emotions, rather than identifying with them [41], [42], [45], [46], is coupled with a decrease in stress perception [45], [47], [48], [49], [50], [51]. Studies have shown that self-other differentiation can be trained and its strengthening can enable a balance between professional distance and human warmth [52]. 

On the other hand, if doctors‘ empathy is reduced to the cognitive dimensions it would mean an unreflectied suppression of emotions – which is coupled in the literature with the onset of exhaustion and burnout [15], [36], [53]. Moreover, emotional distancing has the risk of developing indifference or cynicism toward patients in the long run [21], [54], [55]. A purely cognitive conception of empathy often leads to reliance on learned communication techniques which are considered empathic. Without emotional resonance, they risk becoming feigned empathy which is perceived by the other person as such, and which can lead to dissatisfaction on both sides in the interaction [32], [41].

In summary, it can be concluded that emotional distancing from patients can just as well be the cause of emotional stress, dissatisfaction and burnout among doctors as uncontrolled and unreflected empathic emotions, while reflected and emotion-regulated empathy appears coupled with greater professional satisfaction and burnout prophylaxis [11], [12], [13], [14], [15], [26], [51]. Interventions for promotion of empathy should thus be directed to a reflected and functional dealing with one’s own empathic perception instead of to unspecific increasing of empathy. 

### 1.3. Empathy in medical training

International studies over the past 20 years on changes in empathy during medical training operationalised empathy mostly as a global, cognitive construct, measured in the criteria empathic, not empathic or the extent of empathy [34]. They showed different results (see attachment 1 ): both a significant reduction, especially in the clinical phase with the associated patient contact [56], [57], [58] as well as stability over the years of training [11], [59], [60], [61]. A significant increase was observed less often and ascribed to additional interventions to promote empathy [62], [63], [64]. Medical training thus does not appear to offer an adequate framework in every case to promote this competence. 

### 1.4. Query

The interest of the present study addressed the question whether and in what aspects medical students at the start of their training differ in their empathy from students in the advanced clinical phase of training, in order to gain evidence for generating a conceptualization of teaching contents and interventions to promote empathy during medical training. Starting from a multidimensional empathy concept and study results which delineated specific effects on doctors of the various dimensions of empathy, the hypothesis was formulated that typical empathy profiles can be identified which differ significantly in characterization between the 1^st^ and the 9^th^ semester. 

## 2. Method

### 2.1. Design

In a written survey, medical students at the University of Rostock filled out a self-rating questionnaire. The student’s participation was anonymous and voluntary. The Ethics Commission of the University Medicine Rostock had granted a positive vote. 

### 2.2. Sample

In the study period from 03/2018 to 02/2019, 432 medical students were questioned at the end of obligatory courses. The response rate was 95.6%. Thus, a sample of 413 students was available for statistical analyses, of whom 192 were in the 1^st^ semester and 221 in the 9^th^ semester. 63.9% of the sample were women. 

### 2.3. Measuring instrument

The Saarbrücker Personality Questionnaire (SPF-IRI) [65], [66], the German version of the Interpersonal Reactivity Index (IRI), which is frequently used in international education research, was used [47], [65]. This includes both emotional and cognitive components, as well as emotion regulation [46]. It thus accorded with the multidimensional understanding of empathy in this study. 

The SPF consists of four items for each of the dimensions *perspective taking (PT), empathic concern (EC), fantasy scale (FS)* and *personal distress (PD)*.

The scale *perspective taking (PT)* measures the cognitive capability of seeing a situation from the perspective of another person [67], [65]. The *fantasy scale (FS)* covers the tendency to enter into the emotional world of fictional figures in films or novels and is considered a measure of the strength of emotionality [65] and expression of general empathic reaction readiness [21].The scale empathic concern (EC) measures the tendency to other-oriented feelings – like warmth, sympathy and care – for people in emotional emergency situations [67], [65]. The scale *personal distress (PT)*, on the other hand, is intended to measure the experience of self-focussed feelings, such as the feeling of discomfort, pressure or restlessness in the face of the negative experiences and feelings of others [67], [65] and makes statements about self-other-differentiation and emotion regulation [68], [69]. 

The 16 items are answered on a 5-point Likert scale (1=“doesn’t apply at all”; 5=“applies completely”). 

### 2.4. Statistical procedures

To be able to compare the two study semesters to one another, we used descriptive procedures, mean (*M*), standard deviation (*SD*) as well as inferential statistical analyses. 

Following Altmann’s [45] suggestion, the recorded data were arranged using cluster analysis to form as similar groups as possible with high intracluster-homogeniety and low intercluster-homogeniety, resulting in identification of characteristic subgroups of students with typical, individually differentiable empathy profiles (combination of the four empathy dimensions) [70], [71]. For this, we used two cluster analysis procedures [45], [72], hierarchical cluster analysis [73] and k-means clustering [74]. Then the cluster affiliation was inferential-statistically examined in dependence on semester and gender. 

The significance level was set at 5% for all analyses performed. The Statistical Package for Social Sciences Version 24 (SPSS 24) was used for all analyses. Compliance with the requirements for the statistical procedure was tested before proceding. 

## 3. Results

### 3.1. Empathy profiles in 3 clusters

Complete data sets were available from 391 students for two-stage clustering. In the first step, the number of clusters and a baseline classification were determined by hierarchical clusters. Stepwise fusion of the assignment elements based on Euclidian distance and error-square increase resulted in three to five clusters which were statistically appropriate. This solution was optimized using the k-means method in the second step. Three meaningful, independently delineated clusters could be identified by non-hierarchical new arrangement of the original classification. The numerical distribution of the students to clusters 1 and 2 was optimally equal and was acceptable for cluster 3, since none of the groups was double the size of the others. 

**Figure 1:**
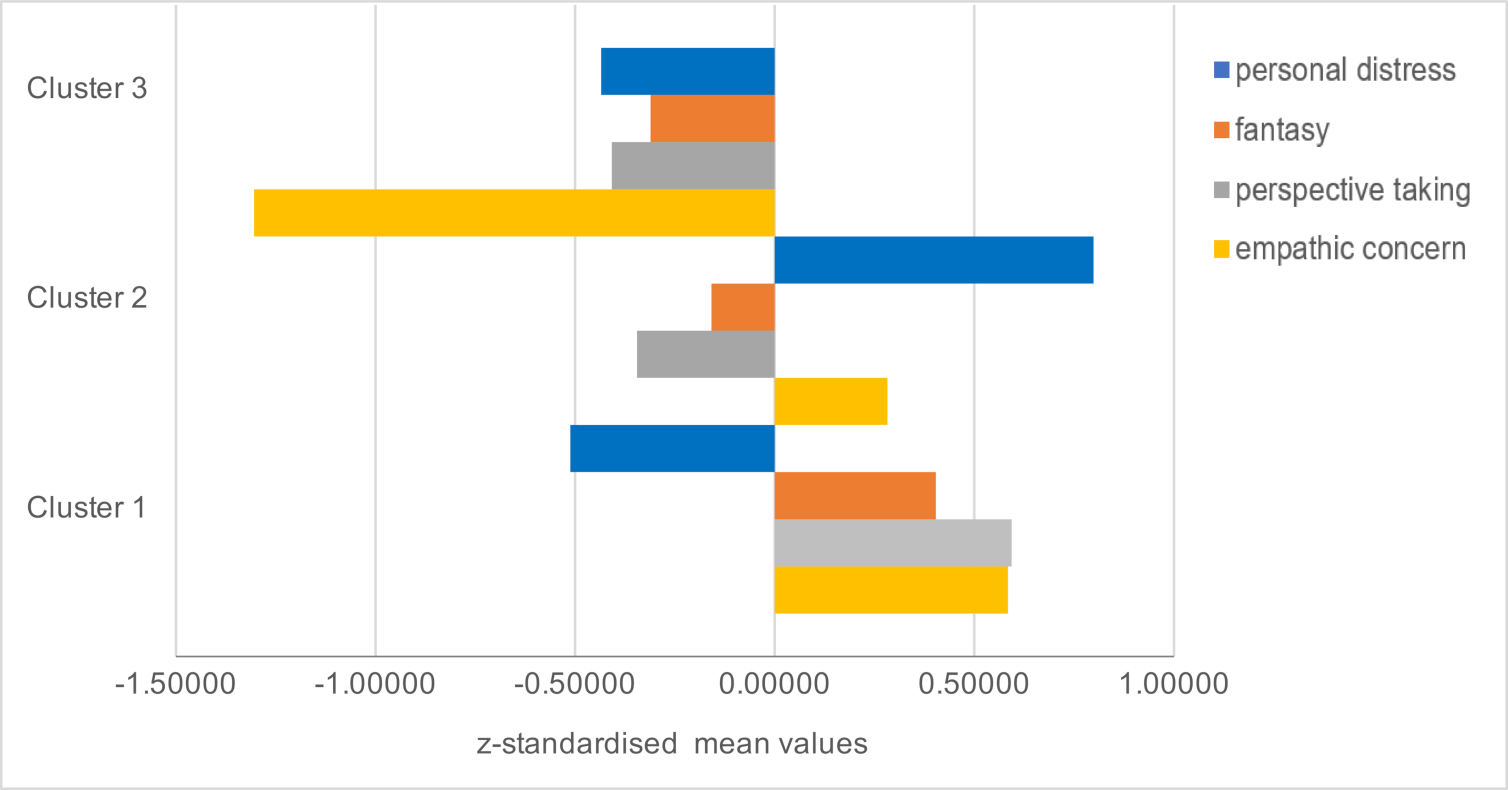
Empathy-profiles in three clusters Cluster 1: Reflected and functional empathy, cluster 2: Unreflected and burdensome empathy, cluster 3: Distancing and avoidance

With respect to the four dimensions on which the cluster formation was based, three empathy profiles could be described for content (see table 1 (Tab. 1) and figure 1 (Fig. 1)).

Compared to the other clusters, cluster 1 showed the lowest values in *personal distress (PD)*, coupled with the highest values in *empathic concern (EC), perspective taking (PT)* and *fantasy scale (FS)*, whereby the values in* perspective taking (PT)* were somewhat higher than in *empathic concern (EC)*. This permits assumption of reflected and functional empathy. 

Compared to the other clusters, cluster 2 showed the highest degree of *personal distress (PD)*, a slightly elevated degree of *empathic concern (EC) *and a rather low degree of *perspective taking (PT)* and *fantasy scale (FS)*. This indicates unreflected, burdensome empathy. 

Cluster 3 is characterized by lower-than-average values in all four dimensions, whereby the low degree of *empathic concern (EC)* was particularly conspicuous. This indicates distancing and avoidance of empathy-promoting interactions.

### 3.2. Comparison of the empathy profiles in dependence on semester and gender

Arrangement of the cluster composition by the demographic data gender, age and semester is shown in table 2 (Tab. 2). 

The cluster affiliation correlated significantly with the variables Semester, χ^2^(2)=19.0, p<.001, and gender, χ^2^(2)=19.06, p<.001, but not with the variable age χ^2^(4)=9.35, p=.053. Cluster 1 consisted of 70.7% women, 55.6% of the persons were students in the 1^st^ semester. In cluster 2, 68.5% of the persons were women and 67.5% were in the 9^th^ semester. Cluster 3 consisted of 55.3% men and 54.2% students in the 1^st^ semester.

### 3.3. Comparison of the semesters in relation to the empathy profiles

**Figure 2:**
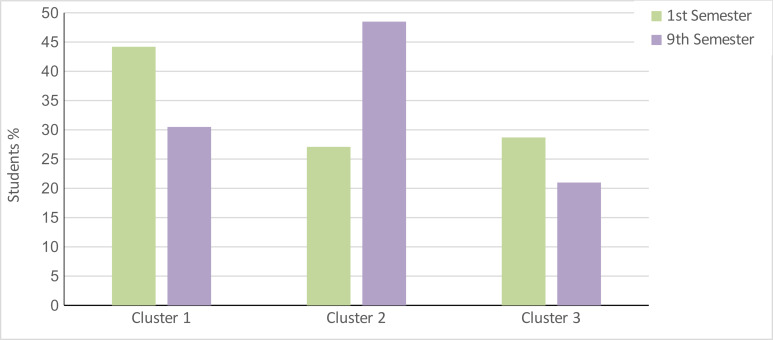
Comparison of the clusters by semester Cluster 1: Reflected and functional empathy, cluster 2: Unreflected and burdensome empathy, cluster 3: Distancing and avoidance

Comparison of the 1^st^ and 9^th^ semesters based on cluster affiliation showed the following result (see figure 2 (Fig. 2)): In the 1^st^ semester, cluster 1was the most marked, followed by cluster 3 and cluster 2. In the 9^th^ semester, most of the students belonged to cluster 2, followed by cluster 1 and cluster 3. 

**Figure 3:**
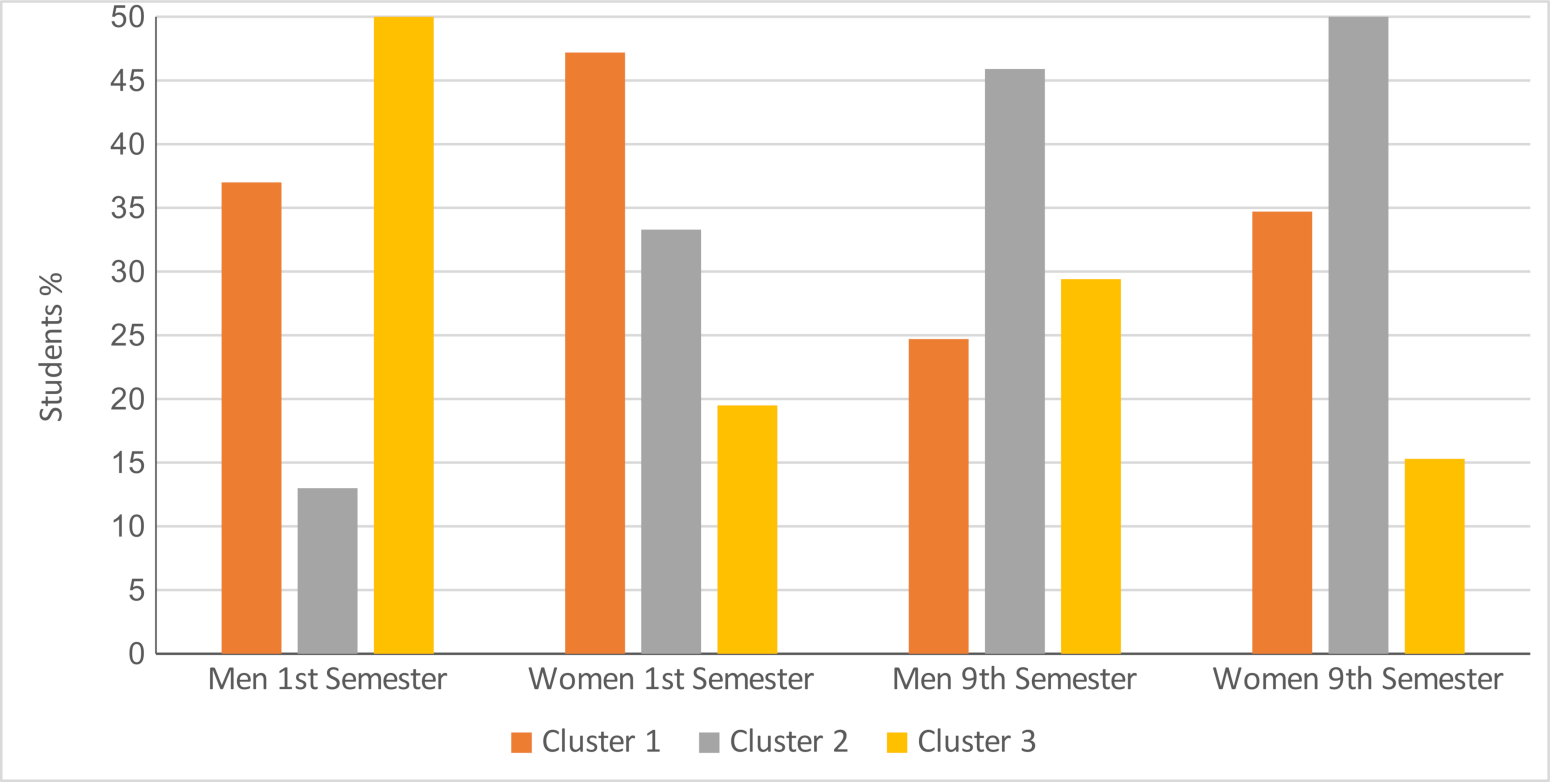
Comparison of 1^st^ and 9^th^ semester by cluster assignment (men and women) Cluster 1: reflected and functional empathy, cluster 2: unreflected and burdensome empathy, cluster 3: distancing and avoidance

The additional examination of the influence of gender showed clear differences between male and female students with respect to their cluster affiliation (see figure 3 (Fig. 3)).

## 4. Discussion

Based on studies of the interaction of the four dimensions *empathic concern (EC), fantasy scale (FS), perspective taking (PT)* and *personal distress (PD)* measured with the SPF, the hypothesis was formulated that, based on the recorded values, typical empathy profiles can be identified which differ in characterization between the 1^st^ and 9^th^ semester. This hypothesis could be confirmed. 

The two stage clustering resulted in three clusters with empathy profiles which could be differentiated meaningfully from one another based on the underlying dimensions:

1. reflected, functional empathy,
2. unreflected, burdensome empathy and
3. distancing and avoidance.

There were clear differences in the affiliation with the identified empathy profiles between the students at the start of their training and those in the 9^th^ semester. 

The capability of reflected, functional empathy (cluster 1), which was the largest cluster in the 1^st^ semester, appeared less pronounced in both genders in the 9^th^ semester. Studies have shown that the combination of high values in *empathic concern (EC)* and *perspective taking (PT)* with concurrent low values in *personal distress (PD)* is coupled in doctors with a high compassion satisfaction [13], professional satisfaction and effectiveness [12], [55] and only rarely with problems in the interaction with patients [21]. Overall, this empathy profile appears to meet the needs of both patients and doctors. 

Students in the 9^th^ semester showed a stronger tendency to unreflected, burdensome empathy (cluster 2) than in the 1^st^ semester, whereby the difference among male students was particularly conspicuous. The high values in *personal distress (PD), slightly elevated values in empathic concern (EC)* and rather low levels in *perspective taking (PT)* and *fantasy scale (FS)* indicate unreflected processes of emotional mirroring and permit the assumption of dysfunctional emotion regulation and deficient self-other-differentiation [41], [45], [51], [75], [76]. This profile is likely more often coupled with compassion fatigue, chronification of reactive stress and increased burnout risk than with efficiency and compassion satisfaction [13], [21], [55]. In patient contact, high emotional distress can lead to pseudoempathic reactions or other termination of the empathic interaction [41], [45] and thus to negative effects for both patient and doctor. 

An increasing tendency during the study to distancing, as has been observed as an avoidance strategy to cope with increasing burden [77] in other studies, could not be confirmed. However, the proportion of students who tended toward distancing and avoidance in studies requiring empathy (cluster 3) – that is, who show little readiness to perspective taking and empathic concern – was alarmingly high at more than 20% in both semesters. This profile can lead to cynicism and indifference of the student toward patients and in the long term to professional dissatisfaction and a reduced experience of efficiency [21], [55] Moreover, it bears the danger of impaired interaction, coupled with lower diagnostic accuracy, negative effects on therapy results and greater emotional stress and lower satisfaction for the patients [5]. 

The study results indicate that the majority of students in the 9^th^ semester are not lacking empathy but primarily an adaptive capability to regulate their emotions in situations requiring empathy – coupled with the danger for patient outcome and their own well-being. The capability and willingness to react with empathy toward patients coupled with the capability of appropriately regulating their own rising emotions, thus protecting themselves against emotional overburdening appears to be present in only one-third of the students. 

Various causes are plausible for this: a reduction of existing regulation capability due to the generally high workload of the students coupled with the experience of stress and time pressure in the clinical setting [13], [21], a lack of role models and a lack of positive examples in dealing with emotions [77], [78], [79], subjective empathy concepts with dysfunctional professional empathy expectations as well as a lack of knowledge about empathic processes and the efficacy of emotion regulation [51], prioritisation of biomedical knowledge [79] and a lack of space and support for reflection and coping with the emotional challenges of the medical profession. 

## 5. Strengths and limitations

This study is, to our knowledge, the first to identify and investigate the empathy profiles of medical students in order to record empathy in its complex multidimensionality and thus be able to point out differentiated action options.

The essential limitation of this study is its cross-sectional design, which does not enable conclusions about causality, Moreover, the empathy differences between the semesters might also be attributable to cohort effects. Based on the same selection procedure for medical training and the low age differences, we consider this bias to be negligible. Furthermore, the use of a self-rating scale bears the danger of result distortion from socially desirable or status-dependent response behaviour. 

In-depth longitudinal studies should investigate the changes in doctors‘ empathy over the course of training and beyond. Using a mixed-method approach could offset the influence of existing teaching formats in order to enable development of effective didactic concepts. Moreover, the extent to which various interventions to promote emotion regulation influence the student’s individual empathy should be investigated. 

## 6. Conclusion

Doctors’ empathy is understood in the framework of this study as an indispensable basis for the trusting doctor-patient relationship which is necessary on both sides 

Two-thirds of the 9^th^ semester students tended toward dysfunctional empathy or distancing, and thus presumably had inadequate adaptive emotional regulation to meet the emotional challenges of studies and patient contact. The result underlines the necessity of continuous implementation of teaching offers on strengthening empathy in the framework of a longitudinal communication curriculum. 

These must not, however, be limited to learning communication techniques, but must be coupled with instructed reflection on one’s own actions, thoughts and feelings if they are to remain effective over time. Medical students should intensively address their subjective concepts of empathy and possible dysfunctional professional empathy expectations. For this, they need space and support for reflection and coping with the emotional challenges of their profession, trained, competent and empathic instructors, as well as positive examples in dealing with emotions. The self-other differentiation is considered a key resource in preventing dysfunctional empathic reactions. Since strengthening this can enable a balance between professional distance and human warmth, attention should be paid to it during the course of medical training. Greater inclusion of scientific concepts about empathy processes, emotion regulation and neurobiological empathic processes in existing medical teaching formats in various disciplines can underline the relevance of empathy as a basic competence for a medical doctor. Medical training must confront these challenges if it wants to train competent and healthy doctors. 

Training of doctors’ empathy should cover this in its multidimensionality and include cognitive, emotional, emotion-regulating and communication-oriented aspects. 

## Notes

### Authors’ ORCIDs

- Susanne Schrötter:0009-0004-5811-1392
- Peter Kropp: 0000-0001-6469-4740
- Britta Müller: 0000-0001-8759-2667

### Authors’ contributions

The authors contributed to the preparation, organisation and performance of the study, as well as to its evaluation and to writing the present manuscript.

- Susanne Schrötter: Conception, preparation, performance of the study, data evaluation and data interpretation, writing the manuscript
- Peter Kropp: Performance of the study, discussion of the manuscript
- Britta Müller: Data evaluation and data interpretation

Both co-authors have read the manuscript and agree to its publication in the present form.

### Statement on the ethical standard

Students’ participation was anonymous and voluntary. They signed a written statement to this effect. The Ethics Commission of University Medicine Rostock granted a positive vote (registration number: A 2018-0052).

## Competing interests

The authors declare that they have no competing interests. 

## Supplementary Material

Empathy of medical students during the course of training – international study results

## Figures and Tables

**Table 1:**
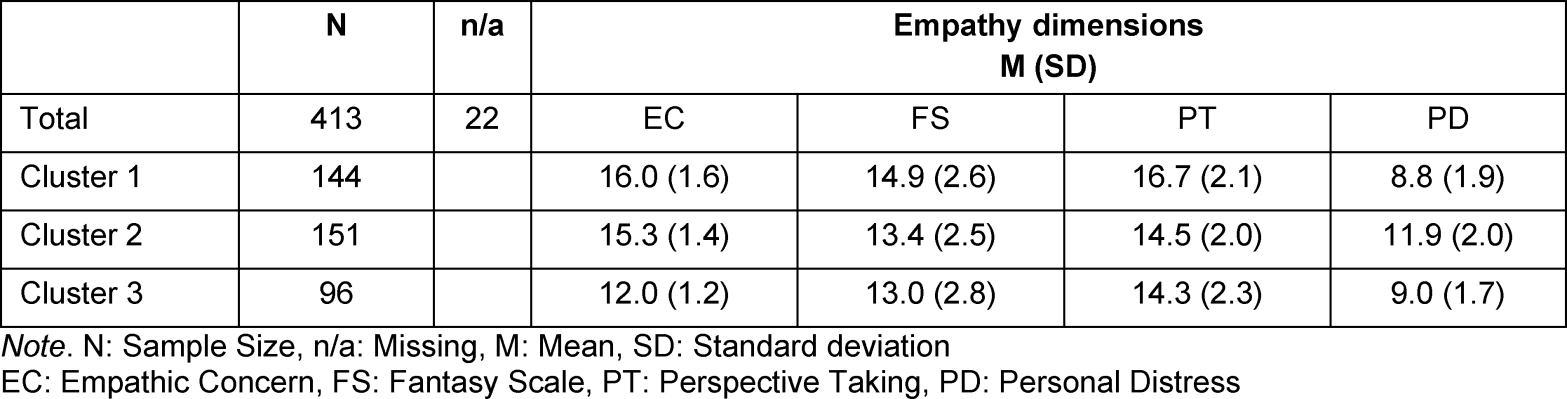
Empathy profiles in three clusters

**Table 2:**
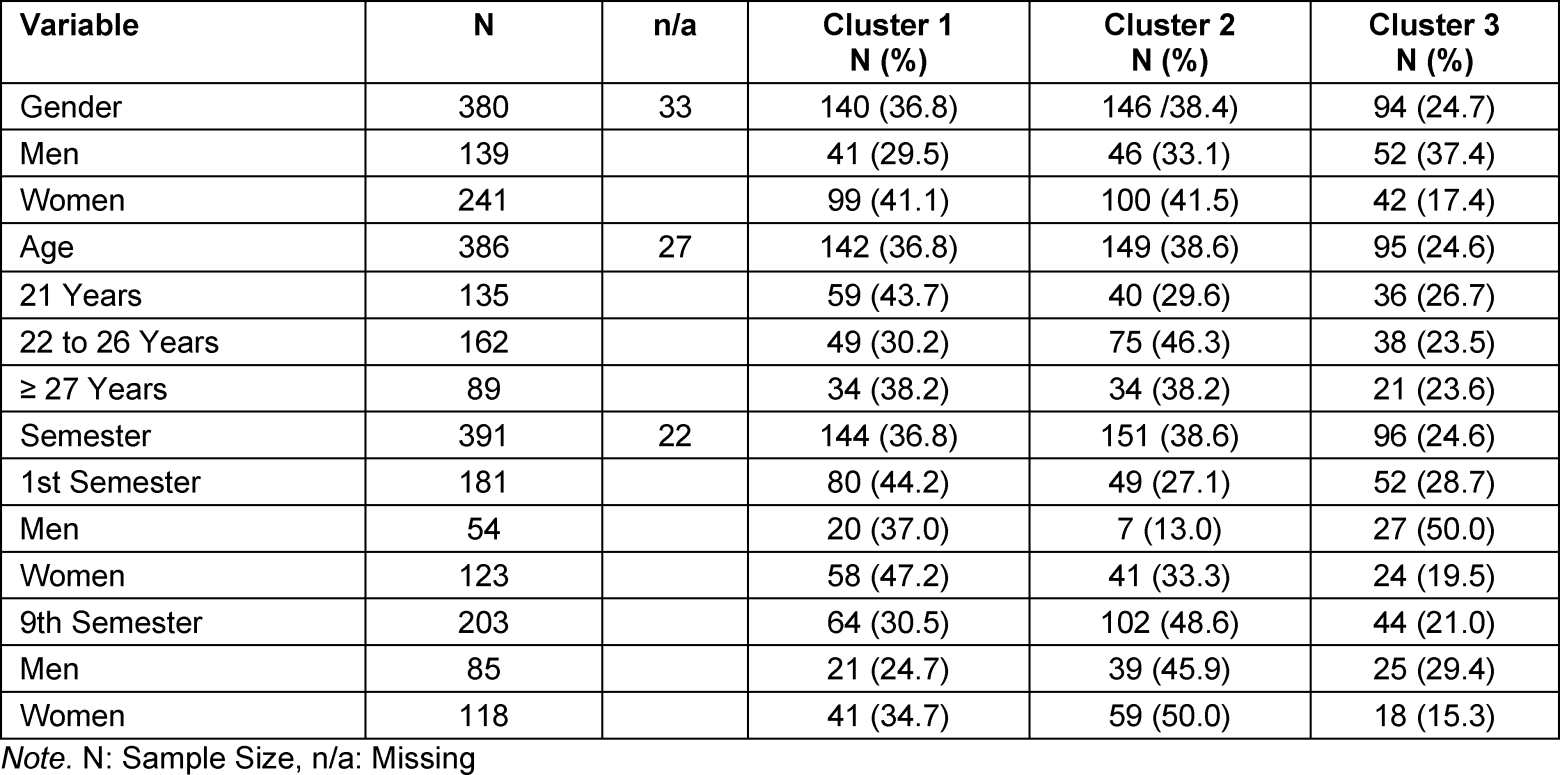
Cluster assignment by age, gender and semester
