# Supplementary material for: Comparison of empathy profiles of medical students at the start and in the advanced clinical phase of their training
Source: GMS J Med Educ. 2024 Feb 15;41(1):Doc7. doi: 10.3205/zma001662 (PMC10946216; doi:10.3205/zma001662)
Supplement: Empathy of medical students during the course of training – international study results [file JME-41-7-s-001.pdf]

Attachment 1: Empathy of medical students during the course of training – international study results

Authors	Year	Country	Sample (N)	Design	Measuring instrument	Significant differences in empathy over time	Development trend	Other significant results
Hojat et al. (N)	2004	USA	125	L	JSPE	Study progress correlates negatively with empathy (Begin 3rd SY > End 3rd SY)	--	No significant influence of gender and age
Chen et al. (N)	2007	USA	658	Q	JSPE	Study progress correlates negatively with empathy (1st SY > 4th SY)	--	Empathy: Women > Men, Patient-oriented course > Non-patient-oriented course
Austin et al. (N)	2007	UK	273	Q	JSPE	Preclinic: Women: decrease, Men: increase, later relatively stable	-- / =	Empathy: Women > Men
Hojat et al. (N) (F)	2009	USA	456	L	JSPE	Study progress correlates negatively with empathy (3rd SY > 4th/5th SY)	--	Empathy: Women > Men, Patient-oriented course > Non-patient-oriented course
Kataoka et al. (F)	2009	Japan	400	L	JSPE	Study progress correlates positively with empathy (6th SY > 1st SY)	+	Empathy: Women > Men
Roh et al.	2010	Korea	493	Q	JSPE	Study progress correlates positively with empathy (4th SY > 1st, 2nd, 3rd SY)	+	No significant gender-specific differences
Chen et al.	2010	USA	322	Q	JSPE OSCE	Study progress correlates negatively with empathy (2nd SY > 3rd SY)	-- / +	Study progress correlates positively with observed empathy in the OSCE (3. SY > 2. SY)
Rahimi-Madiseh et al.	2010	Iran	181	Q	JSPE	None	=	No significant gender-specific differences
Magalhães et al. (F)	2011	Portugal	476	Q	JSPE	Study progress correlates positively with empathy (6th SY > 1st SY)	+	Empathy: Women > Men, No significant correlation with course focus
Loureiro et al. (F)	2011	Portugal	81	Q	JSPE	Study progress correlates positively with empathy (over 6 months in 1st SY)	+	Positive correlation: Empathy and participation in the medical psychology course
Gonullu & Oztuna	2012	Turkey	752	L	JSPE	Study progress correlates negatively with empathy	--	Empathy: Women > Men

Authors	Year	Country	Sample (N)	Design	Measuring instrument	Significant differences in empathy over time	Development trend	Other significant results
Chen et al.<br>(F)	2012	USA	1162	L	JSPE	Study progress correlates negatively with empathy<br>(Preclinic > Clinic)	--	Empathy:<br>Women > Men,<br>Patient-oriented course > Non-patient-oriented course,<br>With high baseline values, lower reduction
Hong et al.	2012	Japan	334	L	JSPE	Study progress correlates positively with empathy<br>(4th SY > 3rd SY > 1st SY)	--	No significant influence of age and gender on empathy, culture-dependent differences
Magalhães et al.	2012	Portugal	350	Q	JSPE<br>NEO-FFI	No information		Positive correlations:<br>Empathy and Big Five (FFM): Agreeableness and openness to experiences,<br>Agreeableness and patient-oriented course
Shariat & Habibi	2013	Iran	1187	Q	JSPE	Study progress correlates negatively with empathy	--	Empathy:<br>Women > Men
Costa et al.<br>(F)	2013	Portugal	77	L	JSPE<br>NEO-FFI	None<br>(latent growth model)	=	Empathy:<br>Women > Men<br>Positive correlations: Empathy and Big Five (FFM): Agreeableness and openness to experiences
Lim et al.<br>(F)	2013	New Zealand	72	L	JSPE	Study progress correlates negatively with empathy<br>(5th SY > 6th SY)	--	Negative correlation: Self-estimate of empathy and rating by fellow students
Wen et al.<br>(F)	2013	China	820	Q	JSPE	Study progress correlates positively with empathy<br>(4th SY > 3rd SY > 2nd SY > 1st SY)	+	Empathy:<br>Women > Men,<br>American students > Chinese students > Iranian and Japanese students
Hasan et al.	2013	Kuwait	264	Q	JSPE	Study progress correlates negatively with empathy	--	Empathy:<br>Women > Men
Hegazi & Wilson<br>(F)	2013	Australia	404	Q	JSPE	None	=	Empathy:<br>Women > Men,<br>Highest among students with additional courses in personal and professional development
Shashikumar et al.	2014	India	488	Q	JSPE	Study progress correlates negatively with empathy<br>(1st – 3rd SY > 4th SY)	--	Empathy:<br>Women > Men,<br>No significant correlation between empathy and course focus

Authors	Year	Country	Sample (N)	Design	Measuring instrument	Significant differences in empathy over time	Development trend	Other significant results
Youssef et al.<br>(F)	2014	Trinidad and Tobago	699	Q	JSPE<br>TEQ<br>RMET	JSPE / TEQ: Study progress correlates negatively with empathy (1st SY > 5th SY, Downward trend highest in the 3rd SY),<br>RMET: stable (1st SY = 5th SY)	--	Empathy:<br>Women > Men,<br>Significant correlations: JSPE - TEQ<br>No significant correlations: JSPE -- RMET
Park et al.	2015	Korea	5343	Q	JSPE	Study progress correlates negatively with empathy<br>(very small effect strength)	--	Empathy:<br>Women > Men,<br>Western students > Korean students
Santos et al.	2016	Brasil	320	Q	JSPE	None	=	Empathy:<br>Women > Men;<br>Patient-oriented course > Non-patient-oriented course
Papageorgiou et al.	2018	UK	333	L	JSPE	Reduction up to 3rd SY, then renewed increase, but lower values than at start of training	--	Affective empathy:<br>Women > Men,<br>Positive correlations with age
O'Tuathaigh et al.	2019	Ireland	241	Q	JSPE<br>NEO-FFI	None<br>(1st SY = final SY)	=	No significant correlation between empathy and course focus,<br>No significant gender-specific differences<br>Positive correlations: Empathy and Big Five (FFM): Agreeableness and openness to experiences,
Thomas et al.<br>(N)	2007	UK	545	Q	IRI (EC and PT)<br>QoL	None	=	Empathy:<br>Women > Men,<br>Medical students compared to age-matched control group:<br>EC and PT higher,<br>Negative correlations between empathy and burnout (increasing depersonalisation gender-independent with decreased PT and EC, increasing emotional exhaustion with low EC in men, trend toward correlation in women),<br>Positive correlations empathy and well-being (quality of life)
Stratton et al.<br>(N) (C)	2008	USA	64	L	IRI<br>TMMS	Slight fluctuations: EC decreases, PD increases (small effect strengths)	-- / =	No significant gender-specific differences,<br>Significant decrease in TMMS-dimensions:<br>Attention to feelings and mood repair

Authors	Year	Country	Sample (N)	Design	Measuring instrument	Significant differences in empathy over time	Development trend	Other significant results
Quince et al.<br>(F)	2011	UK	1653	L	IRI	PT stable;<br>EC: Men: 1st/2nd SY > 4th/5th SY,<br>(small effect strength)	**= / --**	Empathy:<br>Women > Men
Lourinho & Severo	2013	Portugal	65		IRI<br>Neo-FFI	No information,<br>Single measurement at start of training		Positive correlations:<br>Empathy and Big Five (FFM): Agreeableness and openness to experiences,<br>PD - Neuroticism / Extraversion,<br>PT - Conscientiousness
Rau et al.	2013	Germany	2277	Q	SPF / IRI	None<br>(VK = K = PJ)	**=**	Empathy: Women > Men<br>(lowest difference in PT),<br>Age correlates negatively with PT and FS,<br>FS, PD: Medical students < Norm sample,<br>EC: Medical students > Norm sample
Handford et al.<br>(F)	2013	Australia	100	Q	IRI<br>EQ-60<br>RMET	None	**=**	Empathy :<br>Correlations with age, not with SY:<br>Positive: EC,<br>Negative: PD / FS,<br>Positive correlations with Big Five (FFM):<br>EC - Extraversion,<br>PD / FS - Neuroticism
Paro et al.	2014	Brasil	1350	Q	IRI<br>WHOQOL<br>MBI	Relatively stable, low negative correlation	**=**	Gender-specific differences:<br>EC and PD: Women > Men;<br>PT: Women = Men,<br>PT and Gender significant predictors for EC,<br>Correlations Personal accomplishment (MBI): positive with EC and PT, negative with PD,<br>Burnout in all SY (highest at the end of training)
Bratek et al.	2015	Poland	509	Q	IRI	Relatively stable,<br>Women: PT: 6th SY > 1st SY	**= / +**	Empathy: Women > Men,<br>PD: Women = Men,<br>Negative correlations: FS - Age and semester,<br>PD – Age,<br>No significant associations with relationship status and parenthood
van Ryn et al.	2014	USA	4732	Q	IRI<br>JSPE	No information<br>Single measurement at start of training		Attitude toward clinical empathy:<br>Positive correlation with empathy,<br>Further correlations with sociopolitical attitudes, well-being and self-concept

Authors	Year	Country	Sample (N)	Design	Measuring instrument	Significant differences in empathy over time	Development trend	Other significant results
Toto et al.<br>(F)	2015	USA	460	Q	IRI<br>NEO-FFI	1st – 3rd SY stable<br>4th SY > 1st – 3rd SY	**+**	Empathy: Women > Men,<br>Correlation between PT and EC,<br>Positive correlations PT/ EC and Big Five (FFM): Agreeableness and openness to experiences
Krogmann	2018	Germany	240	Q	SPF / IRI<br>NEO-FFI	Study progress correlates negatively with empathy	**-**	Empathy: Women > Men<br>(smallest difference in PT),<br>Age correlates negatively with FS,<br>E, EC, PD: Medical students < Control group<br>Positive correlations empathy and Big Five (FFM): Agreeableness and openness to experiences
Seitz et al.	2017	Austria	77	Q	Qualitativ;<br>Pilot-fragebogen	Study progress correlates negatively with intention to show empathy	**--**	No gender-specific difference in the intention to show empathy
Seitz et al.	2018	Austria	132	Q	GISEB<br>NEO-FFI	Study progress correlates negatively with intention to show empathy<br>(2nd SY > 4th SY)	**--**	No gender-specific difference and no significant influence of personality traits (Big Five) with respect to the intention to show empathy
Triffaux et al.	2019	Belgium	1353	Q	BES	Study progress correlates negatively with empathy	**--**	Affective empathy: Women > Men;<br>Cognitive empathy: Women = Men,<br>Empathy at start of training: Medical students > Control group (Economics students)
Colliver et al.	2010	USA		R*	JSPE (4)<br>IRI (1)<br>BEES (2)	Little or no change	**=**	The contradictory results of the studies reviewed (reduction in empathy) are explained as response bias: in part marked reduction in the response rate
Neumann et al.	2011	USA (8),<br>UK (2),<br>Poland (1)		R**	JSPE<br>IRI<br>BEES	1 Q: stable values<br>otherwise: study progress correlates negatively with empathy<br>(3rd SY > 1st patient contact)	**= /--**	Empathy:<br>Reduction related to increased PD,<br>Patient-oriented course > Non-patient-oriented course,<br>No consistent results with respect to gender-specific differences
Roff	2015	International[1]		LR<br>L / Q	JSPE	Non (latent growth model)	**=**	No information

Authors	Year	Country	Sample (N)	Design	Measuring instrument	Significant differences in empathy over time	Development trend	Other significant results
Ferreira-Valente et al.	2017	International²		R***	JSPE / IRI<br>TEQ /<br>EQ-60<br>RMET	No consistent trends:<br>Q: Stable or increase<br>L: Fluctuations or reduction	**+**<br>**--**<br>**=**	No clear relationship between study results, research design and country,<br>Mostly small effect strengths (16 studies)

Note. (C): in Colliver et al. 2010, (N): in Neumann et al. 2011, (F): in Ferreira-Valente et al. 2017, Q:.Cross-sectional study, L: Longitudinal study, R*: Review: 7 Studies 2000-2008 (3 cross-sectional, 4 longitudinal), R**: Review: 11 Studies 1990-2010 (8 cross-sectional, 3 longitudinal), R***: Scoping Review: 20 Studies 2009-2016 (13 cross-sectional, 8 longitudinal), LR: Literature search, SY: Study Year, VK: Preclinical training phase (1st-3rd SY), K: Clinical training phase (4th-5th SY), PJ: Practical year, OSCE: Objective structured clinical examination**,** IRI: Interpersonal Reactivity Index, JSPE: Jefferson Scale of Physician Empathy, SPF: Saarbrücker Personality Questionnaire, MBI: Maslach Burnout Inventory, WHOQOL: World Health Organization Quality of Life, BEES: Balanced Emotional Empathy Scale, BES: Basic Empathy Scale, NEO-FFI: NEO-Five-Factor-Inventoryr, FFM: Five-Factor Model of Personality, GISEB: General Intention to Show Empathic Behavior, TMMS: Trait Meta-Mood Scale (emotional intelligence measure), QqL: Quality of Life (various measuring tools), EQ-60: Empathy Quotient Questionnaire, TEQ; Toronto Empathy Questionnaire, RMET: Reading the Mind-in-the Eyes Test, E: Total Empathy-Score, EC: Empathic concern, FS: Fantasy scale, PT: Perspective taking, PD: Personal distress, international¹: Japan, South Korea, China, Kuwait, India, Iran, UK, USA, Australia, Brasil, Portugal, Columbia, Dominican Republic, International²: USA, UK, Poland, Portugal, Spain, Australia, Japan, New Zealand, Brasil, China, Venezuela, Trinidad and Tobago, Dominican Republic.
